# A new player in psoriatic arthritis: a JAK inhibitor

**DOI:** 10.31138/mjr.28.4.210

**Published:** 2017-12-22

**Authors:** Lazaros I. Sakkas

**Affiliations:** Department of Rheumatology and Clinical Immunology, Faculty of Medicine, School of Health Sciences, University of Thessaly, Larissa, Greece

**Keywords:** JAK, inhibitors, psoriatic arthritis, psoriasis

Psoriatic arthritis (PsA) is an inflammatory arthritis in patients with psoriasis (Pso). Pso affects 2-4% of the general population while PsA affects 65% of Pso patients if one uses even regional imaging modalities.^1,2^ PsA can involve peripheral joints and spinal joints, causing oligoarticular or polyarticular arthritis and spondyloarthropathy, but also affects various tissues causing enthesitis, dactylitis, and uveitis. A radiographic characteristic of PsA, not found in rheumatoid arthritis (RA), is that it can cause bone erosions and bone formation at various joint sites. The pathogenesis of the disease is incompletely understood. Nevertheless, inflammatory cytokines, such as interleukin (IL)-12, IL-17, interferon (IFN)γ, IL-23, and IL-22, and cells of the adaptive immunity and innate immunity are implicated. Moreover, the elevated cytokines of the IL-23/IL-17 axis with IL-17 and IL-22 of the axis having erosive and bone-forming actions, respectively, suggest that PsA and psoriasis may be the same disease.^3^

**Figure 1.**
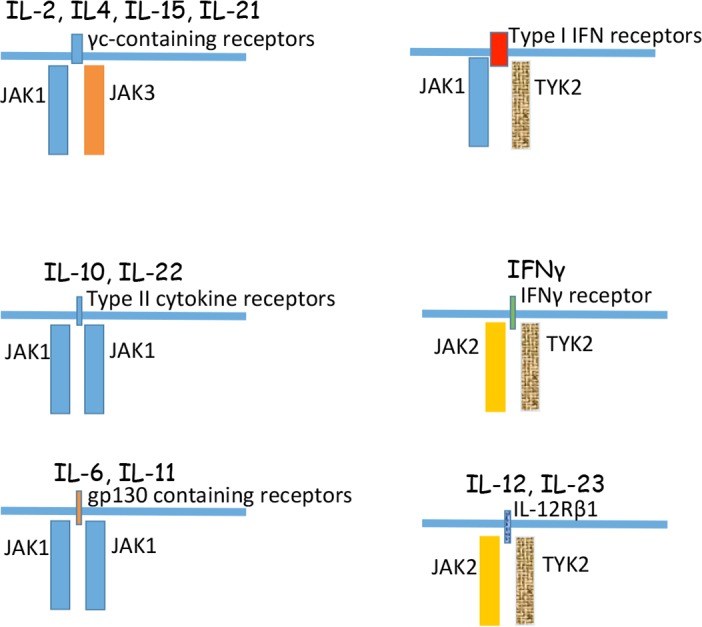
Activation of cytokine pathways via JAKs.

Recent recommendations for the treatment of PsA include non-steroidal anti-inflammatory drugs (NSAIDs), conventional synthetic disease-modifying antirheumatic drugs (DMARDs), phosphodiesterase 4 inhibitor (Apremilast), and biologicals, including tumour necrosis factor (TNF) inhibitors (TNFi), IL-17 inhibitors (secukinumab) and IL-12/IL-23 inhibitors (ustekinumab).^4,5^ A Janus kinase (JAK) inhibitor, tofacitinib, an oral agent approved for the treatment of RA, was used in either TNFi-naïve or TNFi-experienced PsA patients in two randomized controlled trials published in the October issue of New England Journal of Medicine.^6,7^ The Janus family kinases, JAK1, JAK2, JAK3 and TYK2, are cytoplasmic non-receptor protein kinases. As such, the phosphorylate tyrosine leave residues of their protein substrates. JAKs as homodimers or heterodimers bind to transmembrane receptors, activated by extracellular cytokines/growth factors, and activate downstream molecules, primarily the signal transducers and activators of transcription (STATs)^8^ (**Figure 1**).

In a 12-month, double-blind, active controlled with adalimumab, placebo-controlled, phase 3 trial, in patients with PsA and inadequate response to conventional synthetic DMARD, tofacitinib was used in a 5-mg twice daily dose, 10-mg twice daily dose, and adalimumab in a 40 mg biweekly dose. At month 3, placebo was blindly switched to 5 mg tofacitinib or 10 mg tofacitinib dose. Overall, there was no differences between the 5 mg dose, the 10 mg dose, and the adalimumab in efficacy across various aspects of the disease (peripheral arthritis, dactylitis, enthesitis, psoriasis) up to 12 months. In particular, at month 3, ACR20 was achieved by 50% in the 5-mg tofacitinib group, 61% in the 10 mg tofacitinib group, and 52% in the adalimumab group, and 33% in the placebo group. ACR50 was achieved by 28% in the 5-mg tofacitinib, 40% in the 10-mg tofacitinib, 33% in the adalimumab, and 10% in the placebo groups. However, during the study, infections - including herpes zoster, and cancer, excluding non-melanoma skin cancer - were increased in patients taking tofacitinib: this should be monitored closely in subsequent studies.^6^ In a second study, a 6-month, randomized, double-blind, placebo-controlled, phase 3 trial of patients with PsA and inadequate response to TNF inhibitors, tofacitinib was used in a 5 mg twice daily dose or 10 mg twice daily dose. At 3 months, placebo was blindly switched to 5 mg or 10 mg dose regimen. The ACR20, ACR50 and ACR70 response at 3 months and 6 months is shown in **Table 1**.^7^ As seen, at 3 months, the ACR50 response was superior in the tofacitinib group compared to placebo, but not the ACR70 response. To have a sense of comparison, in PsA patients with inadequate response to TNF inhibitors, at 6 months, the ACR20 response was achieved by ustekinumab, a IL-12/23 inhibitor, in 35.6% of patients vs 14.5% in the placebo group,^9^ and by secukinumab, an IL-17 inhibitor, in 45.5% of patients vs 14.3% in the placebo group.^10^ Infections, including herpes zoster, major adverse cardiovascular events, neutropenia, lymphopenia, and elevation of transaminases were observed in the tofacitinib group of patients and should be monitored in subsequent studies.

**Table 1.** ACR20, ACR50 and ACR70 response of patients with PsA treated with tofacitinib at 3 months and 6 months.

	**ACR20**	**ACR50**	**ACR70**
**At 3 months**			
Tofacitinib 5 mg	50%	30%	17%
Tofacitinib 10 mg	47%	28%	14%
placebo	24%	15%	10%
**At 6 months**			
Tofacitinib 5 mg	60%	38%	21%
Tofacitinib 10mg	49%	30%	14%

In summary, a new player arrived in the courtyard of PsA. It appears to be effective, but requires vigilance for potential adverse effects.
